# Analysis of long-term mortality after total body irradiation-based and melphalan-based chemotherapy conditioning for acute myeloid leukemia

**DOI:** 10.1007/s00277-023-05318-y

**Published:** 2023-06-22

**Authors:** Isabella Gruber, Oliver Koelbl, Marius Treutwein, Florian Zeman, Wolfgang Herr, Ernst Holler, Matthias Edinger, Daniel Wolff

**Affiliations:** 1 Department of Radiation Oncology, University Hospital Regensburg, Regensburg, Germany; 2 Center for Clinical Studies, University Hospital Regensburg, Regensburg, Germany; 3 Department of Internal Medicine III, University Hospital Regensburg, Regensburg, Germany; 4 Leibniz Institute for Immunotherapy, Regensburg, Germany

**Keywords:** Acute myeloid leukemia, Conditioning, Allogeneic hematopoietic stem cell transplantation, Total body irradiation, Non-relapse mortality, Long-term mortality

## Abstract

Allogeneic hematopoietic stem cell transplantation (allo-HSCT) is a curative treatment option for selected patients with acute myeloid leukemia. Yet, the influence of total body irradiation (TBI)-based conditioning as compared to non-TBI-based conditioning on long-term mortality is unclear. We retrospectively evaluated outcomes after TBI-based (*n* = 91) and non-TBI-based conditioning (melphalan-based, *n* = 248) for 1st allo-HSCT patients transplanted at the University Hospital Regensburg between 1999 and 2020. TBI was performed with an average dose rate of 4 cGy/min. Median follow-up was 8.3 years (interquartile range, 4.8–12.9 years). Cumulative incidence rates of 5-year non-relapse mortality (NRM) were 17% (95% confidence interval, CI, 10–25) and 33% (95% CI, 27–40) after TBI- and non-TBI-based conditioning (*P* < 0.001). Five-year cumulative incidences of relapse (CIR) were 42% (95% CI, 32–52) and 29% (95% CI, 23–35) after TBI- and non-TBI-based conditioning (*P* = 0.030). The 5-year OS was 54% (95% CI, 43–64) and 55% (95% CI, 48–62) after TBI- and non-TBI-based conditioning. Both groups had similar 100-day acute graft-versus-host disease (aGVHD, 43% vs. 40%) and 5-year chronic GVHD (34% vs. 36%). The multivariable regression models found no associations of TBI with the outcomes NRM, CIR, PFS, OS, aGVHD, and cGVHD. TBI was no risk factor for NRM, even including mortality caused by secondary malignancies. NRM was influenced by patient age, advanced disease status, and the use of female donors for male recipients. TBI- and non-TBI-based conditioning appear to be equally effective and tolerable for AML patients eligible for 1st allo-HSCT.

## Introduction

Allogeneic hematopoietic stem cell transplantation (allo-HSCT) is a curative treatment modality for adults with acute myeloid leukemia (AML). However, allo-HSCT is still associated with a high long-term non-relapse mortality rate (NRM). NRM may be attributed to infections, graft-versus-host disease (GVHD), organ toxicity, and secondary malignancies (SMs). One of the most popular myeloablative conditioning (MAC) regimens is CYTBI (twice daily 2 Gy fractions of total body irradiation over 3 days to a total dose of 12 Gy followed by intravenous cyclophosphamide 60 mg/kg × 2 days) [1]. TBI-based myeloablative conditioning (MAC 8–12 Gy) is usually preferred in fit and younger patients (< 50 years of age) with high-risk mutations or cytogenetic risk factors, for whom the combination of TBI and chemotherapy potentiates therapeutic efficacy [2]. Reduced intensity conditioning (RIC) combines lower doses of chemotherapy and/or TBI (doses of ≥ 4 Gy and < 8 Gy) to reduce toxicity while maintaining anti-leukemic effects [2]. It is still unclear how TBI-based conditioning compares to non-TBI-based protocols in patients with AML with respect to long-term mortality. In this retrospective study, we therefore analyzed the long-term mortality (including NRM) of patients after TBI-based conditioning applying a standardized fractionated TBI technique, as compared to non-TBI-based conditioning (melphalan-based protocols) focusing on AML patients and 1st allo-HCST.

## Patients and methods

### Data collection

We retrospectively compared long-term outcomes following TBI-based and non-TBI-based conditioning in patients with AML who received their 1st allo-HSCT at the Department of Hematology of the University Hospital Regensburg between 1999 and 2020. The eligibility criteria for this retrospective analysis included adult patients with primary or secondary AML who underwent their 1st allo-HSCT from matched sibling donors (MSD), matched unrelated donors (MUD), mismatched unrelated donors (MMUD), or haploidentical/mismatched related donors (MMRD) following TBI-based or non-TBI-based conditioning. Non-TBI-based conditioning included FBM (fludarabine, BCNU, melphalan), FTM (fludarabine, thiotepa, melphalan), and FM (fludarabine, melphalan). Source of stem cells were peripheral blood or bone marrow. The exclusion criteria were cord blood transplantation and non-myeloablative (NMA) conditioning (*n* = 11), previous autologous transplantation (*n* = 18), and non-melphalan-based protocols (*n* = 115). In summary, 339 patients were included in this analysis. The choice of conditioning regimen was based on the oncologists’ discretion and dependent on patient age, disease risk, and/or presence of comorbidities. Clinical data were extracted from the medical charts of the Departments of Hematology and Radiation Oncology, University Hospital Regensburg. Transplantation variables included patient age at the time of 1st allo-HSCT, sex, diagnosis, Karnofsky performance score (KPS), hematopoietic cell transplantation-comorbidity index (HCT-CI) as described by Sorror et al. [3], 2017 European Leukemia Net (ELN) genetic risk stratification as described by Döhner et al. [4], disease status before allo-HSCT, stem cell source, intensity of conditioning regimen, recipient and donor characteristics (donor age, HLA compatibility, sex match, cytomegalovirus serostatus, ABO blood group compatibility), GVHD prophylaxis, and the use of antithymocyte globulin (ATG). Variables related to outcome were the cumulative incidences of relapse (CIR), NRM, grade II–IV aGVHD (acute graft-versus-host disease), cGVHD (chronic graft-versus-host disease requiring immunosuppressive treatment), OS, PFS, and causes of death including mortality caused by secondary malignancies (SMs). The Clinical Cancer Registry at the Tumor Center Regensburg and local Viability Statistics Registration Offices were contacted to confirm the survival status before statistical analysis for patients with long site visit intervals resulting in data completeness of 100%. Data closing was April 2021. The local Ethics Board of the University of Regensburg approved this study (Number 20–1810-101).

### TBI

From 1999 to 2013, two Siemens Primus linear accelerators (Siemens Medical Systems, Inc., Concord, CA) were used for TBI, and from 2013 to 2020 two linear accelerators of type Elekta Synergy™ with an Agility™ head (Elekta Ltd, Crawley, UK). We proved clinically good dose distributions and similar parameters with both linear accelerators [5]. All patients received 6 megavoltage (MV) photon beams. We adopted a twice-daily fractionation and a minimum of 6 h between the fractions. Patients were lying down on a couch at the floor level in supine and prone positions to extend the source-to-skin distance. A plate of Makrolon® polycarbonate of 1 cm thickness was placed on a stand above of the patient to neutralize the skin sparing by the buildup effect. The low diameter in the neck region was compensated by using a bolus of plastic modeling mass. Eight rotational arcs were used per patient position. The average time to deliver each fraction was 50–60 min per side (supine and prone), and the average dose rate to the total body was 4 cGy/min. Additional fixed beams were used in cranial and caudal direction to compensate for the effects of inverse square variation with increasing distance. Two individual lung shields of MCP96 of calculated thickness were designed in case of doses > 8 Gy to reduce the total dose to the center of the lung to 3.5 Gy in supine and prone positions (total dose of 7 Gy). Radio-oncologists contoured two individual lung blocks for each patient on a CT scan with a 1 to 2 cm margin between the edge of the lung on the CT film and the edge of the block. Lung blocks were tailored to avoid shielding of the vertebrae. MV imaging verified the shielding positions. Areas of the chest wall that were shielded by the blocks were supplemented once a day with electron beams to achieve the full dose to the thoracic walls. The electron fields delivered a supplemented dose of 5 Gy for 12 Gy regimens. In vivo dosimetry was used to verify the dose delivery on several points on the patient’s body, demonstrating the uniformity of the dose distribution [5].

### Definitions and statistical endpoints

The primary endpoints were cumulative incidences of NRM with relapse considered a competing event. Secondary endpoints were cumulative incidences of relapse (CIR), grade II–IV aGVHD, cGVHD (requiring immunosuppressive treatment), PFS, OS, and causes of death including SMs. All times to the endpoints were calculated from the date of allo-HSCT (day 0). NRM was defined as death from any cause in the absence of prior relapse of the initial AML, with relapse considered a competing event. Relapse was defined as manifest hematologic relapse requiring treatment. Isolated mixed chimerism or molecular detection of minimal residual disease (MRD) not requiring intervention was not regarded as relapse. For CIR, death from NRM was counted as a competing event. Acute GVHD and cGVHD were defined according to described standard criteria [6, 7]. Acute GVHD was classified as clinically significant at grade II–IV aGVHD. The cumulative incidence of grade II–IV aGVHD was estimated considering death or relapse without grade II–IV aGVHD as a competing event. For the cumulative incidences of cGVHD (requiring immunosuppressive treatment), relapse or death without prior cGVHD (requiring immunosuppressive treatment) was counted as a competing event. PFS was defined from the date of allo-HSCT to the date of relapse, progression, or death from any cause. If patients were transplanted with active disease and did not reach complete remission after allo-HSCT, the date of relapse was defined as day 0. OS was defined as the time from allo-HSCT to the date of death by any cause. If a patient was event-free for all of the endpoints, the patient was censored at the last date of follow-up with confirmation of being event-free. To adjust for any potential bias derived from imbalanced patient characteristics between TBI-based and non-TBI-based conditioning, multivariable regression analysis was used. Covariates were ELN 2017 risk stratification, diagnosis, disease status, HCT-CI, patient age, conditioning intensity, KPS, donor type, graft source, sex match, donor age, donor/recipient CMV status, and the use of ATG. Standardized consensus definitions of hematopoietic recovery, graft failure, and donor chimerism were used [8]. Peripheral blood PCR-based chimerism analyses were performed on a regular schedule. Full donor chimerism was defined as 99% or greater donor chimerism. Patients were censored from the engraftment analysis if they died or had persistent leukemia/early relapse within the first 28 days after allo-HSCT. Causes of death are subdivided in a hierarchical manner with descending priority: AML, GVHD, infection, and other causes [9]. Deaths from AML include cases with disease progression or relapse after allo-HSCT. Deaths from GVHD include cases with acute and/or chronic GVHD on active treatment without infection and without evidence of disease progression or relapse after allo-HSCT. Deaths from GVHD include cases with active treatment of GVHD and documented infections contributing to death (GVHD is severe enough to cause death even if infection did not occur). Death from infection includes infections causing death without evidence of disease progression, relapse, or GVHD. Death from infection includes cases of infections with a history of GVHD that had resolved and was not treated at the time of infection. Infectious deaths are analyzed as total (bacterial, fungal, parasitic, viral, mixed, and unknown). Other causes of death include secondary malignancies, graft failures, accidents, suicides, hemorrhage, and thrombosis without evidence of disease progression or relapse after allo-HSCT, and without infection or GVHD.

### Statistical analysis

Transplant-related characteristics for the TBI and non-TBI group are presented as median and interquartile range (IQR) for continuous variables and as absolute and relative frequencies for categorical variables. The Mann–Whitney *U*-test was used for comparisons of continuous variables, and the chi-square test of independence for categorical variables. The time-to-event endpoints CIR, NRM, grade II–IV aGVHD, and cGVHD were analyzed using uni- and multivariable Fine and Gray proportional hazard regression models to account for the respective competing events. The proportional hazard assumption of the Fine and Gray models was tested by using rescaled Schoenfeld-type residuals. PFS and OS were analyzed by uni- and multivariable Cox proportional hazard regression models. Hazard ratio (HR) and 95% confidence interval (95% CI) are presented as effect estimate. Cumulative incidence functions at fixed time points were compared using the method with Gaynor’s variance proposed by Chen et al. [10]. Median follow-up time was estimated by the reverse Kaplan–Meier method. All *P*-values were two-sided and *P*-values < 0.05 were considered significant. Statistical analysis was performed using SPSS 26.0 (SPSS Inc., Chicago, IL, USA) and R, version 4.1.2 (R Core Team. R: a language for statistical computing. 2014. The R Foundation for Statistical Computing, Vienna, Austria).

## Results

### Patient and transplantation characteristics

Table 1 summarizes patient, disease, and transplant characteristics of the included patients (*n* = 339). Patients received their 1st allo-HSCT for de novo/primary AML (*n* = 227) or secondary AML (*n* = 112) after TBI-based conditioning (*n* = 91) or non-TBI-based conditioning (*n* = 248) with peripheral blood (*n* = 314) or bone marrow (*n* = 25) as stem cell source. Median follow-up time of TBI patients was longer in comparison to patients of the non-TBI group (12.6 years vs. 6.7 years; *P* < 0.001). Patients of the TBI group were younger at the time of 1st allo-HSCT (median 41.6 years, IQR, 32.2–50.7) compared to patients of the non-TBI group (median 56.8 years, IQR, 48.9–63.0; *P* < 0.001), and had more de novo/primary AML (75.8% vs. 63.7%; *P* = 0.038) and a lower HCT-CI (score 0: 44.0% vs. 26.2%; *P* < 0.001). All conditioning regimens are summarized in Table 2.

**Table 1:** Transplant characteristics of TBI-based conditioning and non-TBI-based conditioning

	Total (*n* = 339)	TBI-based conditioning (*n* = 91)	Non-TBI-based conditioning (FBM, FTM, FM) (*n* = 248)	*P*-value
Follow-up, years				< 0.001
Median (IQR)	8.3 (4.8–12.9)	12.6 (10.0–15.6)	6.7 (4.0–11.0)	
Patient age at 1st allogeneic hematopoietic stem cell transplantation, years				< 0.001
Median (IQR)	54.4 (43.6–61.0)	41.6 (32.2–50.7)	56.8 (48.9–63.0)	
Sex, *n* (%)				0.531
Male	205 (60.5%)	58 (63.7%)	147 (59.3%)	
Female	134 (39.5%)	33 (36.3%)	101 (40.7%)	
Diagnosis, *n* (%)				0.038
De novo acute myeloid leukemia	227 (67.0%)	69 (75.8%)	158 (63.7%)	
Secondary acute myeloid leukemia	112 (33.0%)	22 (24.2%)	90 (36.3%)	
Hematopoietic cell transplantation-comorbidity index (HCT-CI), *n* (%)				< 0.001
0	105 (31.0%)	40 (44.0%)	65 (26.2%)	
1–2	117 (34.5%)	36 (39.6%)	81 (32.7%)	
≥ 3	117 (34.5%)	15 (16.5%)	102 (41.1%)	
Karnofsky performance status, *n* (%)				0.284
≥ 80	293 (86.4%)	82 (90.1%)	211 (85.1%)	
< 80	46 (13.6%)	9 (9.9%)	37 (14.9%)	
2017 ELN genetic risk stratification, *n* (%)				0.797
Favorable	54 (15.9%)	13 (14.3%)	41 (16.5%)	
Intermediate	145 (42.8%)	38 (41.8%)	107 (43.1%)	
Adverse	140 (41.3%)	40 (44.0%)	100 (40.3%)	
Disease status at 1st transplantation, *n* (%)				0.172
First complete remission (CR1)	147 (43.4%)	32 (35.2%)	115 (46.4%)	
CR2, first partial remission (PR1)	104 (30.7%)	33 (36.3%)	71 (28.6%)	
> CR2, refractory, active AML	88 (26.0%)	26 (28.6%)	62 (25.0%)	
Donor type, *n* (%)				0.041
Matched sibling donor	100 (29.5%)	33 (36.3%)	67 (27.0%)	
Matched unrelated donor	177 (52.2%)	50 (54.9%)	127 (51.2%)	
Mismatched unrelated donor	53 (15.6%)	7 (7.7%)	46 (18.5%)	
Haploidentical, mismatched related donor	9 (2.7%)	1 (1.1%)	8 (3.2%)	
Stem cell source, *n* (%)				1.000
Peripheral blood	314 (92.6%)	84 (92.3%)	230 (92.7%)	
Bone marrow	25 (7.4%)	7 (7.7%)	18 (7.3%)	
Conditioning regimen, *n* (%)				< 0.001
Standard myeloablative (MAC)	60 (17.7%)	60 (65.9%)	-	
Reduced intensity (RIC)	279 (82.3%)	31 (34.1%)	248 (100%)	
Donor sex, *n* (%)				0.896
Male	230 (67.8%)	61 (67.0%)	169 (68.1%)	
Female	109 (32.2%)	30 (33.0%)	79 (31.9%)	
Donor age, years				0.255
Median (IQR)	39.0 (30.0–47.0)	38.0 (30.0–44.0)	39.0 (30.2–48.0)	
Female donors to male recipients, *n* (%)				0.868
Yes	54 (15.9%)	15 (16.5%)	39 (15.7%)	
No	285 (84.1%)	76 (83.5%)	209 (84.3%)	
Donor/recipient CMV serostatus, *n* (%)				0.175
Negative/negative	137 (40.4%)	43 (47.3%)	94 (37.9%)	
Negative/positive	58 (17.1%)	17 (18.7%)	41 (16.5%)	
Positive/positive	101 (29.8%)	19 (20.9%)	82 (33.1%)	
Positive/negative	43 (12.7%)	12 (13.2%)	31 (12.5%)	
ABO blood group compatibility, *n* (%)				0.671
Ident	147 (43.4%)	36 (39.6%)	111 (44.8%)	
Minor mismatch	83 (24.5%)	23 (25.3%)	60 (24.2%)	
Major mismatch	109 (32.2%)	32 (35.2%)	77 (31.0%)	
Graft-versus-host disease prophylaxis, *n* (%)				0.277
Cyclosporine, MTX	221 (65.2%)	64 (70.3%)	157 (63.3%)	
Cyclosporine, MMF	92 (27.1%)	24 (26.4%)	68 (27.4%)	
Post-Tx cyclophosphamide, tacrolimus, MMF	21 (6.2%)	2 (2.2%)	19 (7.7%)	
Tacrolimus/MMF	5 (1.5%)	1 (1.1%)	4 (1.6%)	
Antithymocyte globulin (ATG), *n* (%)				0.223
Yes	244 (72.0%)	61 (67.0%)	183 (73.8%)	
No	95 (28.0%)	30 (33.0%)	65 (26.2%)	

*TBI*, total body irradiation; *IQR*, interquartile range; *ELN*, European Leukemia Net; *CMV*, cytomegalovirus; *FBM*, fludarabine, BCNU, melphalan; *FTM*, fludarabine, thiotepa, melphalan; *FM*, fludarabine, melphalan

**Table 2:** Conditioning regimens before 1st allogeneic hematopoietic stem cell transplantation

Regimens	*n* (%)
Non-TBI-based conditioning	
FBM (fludarabine, BCNU, melphalan)	174 (51.3%)
Fludarabine 5 × 30 mg/m^2^ (d − 8 to d − 4), BCNU 2 × 150 mg/m^2^ (d − 6, d − 5), melphalan 110 mg/m^2^ on d − 3 (age ≥ 55 years), or melphalan 140 mg/m^2^ on d − 3 (age < 55 years)	
FTM (fludarabine, thiotepa, melphalan)	56 (16.5%)
Fludarabine 5 × 30 mg/m^2^ (d − 7 to d − 3), thiotepa 5 mg/kg (d − 6), melphalan 110 mg/m^2^ on d − 3 (age ≥ 55 years), or melphalan 140 mg/m^2^ on d − 3 (age < 55 years)	
FM (fludarabine, melphalan)	18 (5.3%)
Fludarabine 5 × 30 mg/m^2^ (d − 8 to d − 4), melphalan 140 mg/m^2^ (d − 4)	
TBI-based conditioning	
TBI 8 Gy, cyclophosphamide, fludarabine	42 (12.4%)
TBI 8 Gy (four 2 Gy doses on two consecutive days, d − 8, d − 7), cyclophosphamide 2 × 60 mg/kg (d − 4, d − 3), fludarabine 3 × 30 mg/m^2^ (d − 6, d − 5, d − 4)	
FLAMSA-RIC, TBI 4 Gy, cyclophosphamide	31 (9.1%)
FLAMSA regimen (d − 12 to d − 9), fludarabine 4 × 30 mg/m^2^, HD-Ara-C 4 × 2000 mg/m^2^, amsacrine 4 × 100 mg/m^2^. Reduced intensity conditioning (RIC) regimen after 3 days of rest, TBI 4 Gy on d − 5 (two 2 Gy doses), cyclophosphamide (2 × 40 mg/kg for MRD or 2 × 60 mg/kg for MUD, MMRD, or MMUD) on d − 4 to d − 3, antithymocyte globulin (ATG) 10 mg/kg for MRD or 20 mg/kg for MUD, MMRD, MMUD on d − 4 to d − 2, pDLTs (prophylactic donor lymphocyte infusions) at day + 120 or 30 days after discontinuation of immunosuppression (1–5 × 10^6^ CD3^+^ cells/kg)	
TBI 12 Gy, cyclophosphamide	12 (3.5%)
TBI 12 Gy (six 2 Gy doses, on three consecutive days, d − 7 to d − 5), cyclophosphamide 2 × 60 mg/kg on 2 consecutive days (d − 4, d − 3)	
TBI 8 Gy, fludarabine	6 (1.8%)
TBI 8 Gy (four 2 Gy doses on 2 consecutive days, d − 5 and d − 4), fludarabine 4 × 30 mg/m^2^ (d − 5 to d − 2)	

*MRD*, matched related donor; *MUD*, matched unrelated donor; *MMRD*, mismatched related donor; *MMUD*, mismatched unrelated donor

### Engraftment and chimerism analysis

The TBI and non-TBI groups achieved an absolute neutrophil count (ANC) of > 500 cell/μL (ANC500) at median of 17.0 days (IQR, 14.0–20.0) and 16.0 days (IQR, 14.0–19.7) (*P* = 0.740). Median times for reaching a platelet count of 20,000/μL (PLT20,000) were 16.0 days (IQR, 13.0–21.0) for the TBI group and 18.0 days (IQR, 14.0–24.0) for the non-TBI group (*P* = 0.082). A total of 4 patients had primary graft failure: 2 patients (0.8%) of the non-TBI group and 2 patients (2.2%) of the TBI group (*P* = 0.293). One patient (1.1%) of the TBI group and 5 patients (2.0%) of the non-TBI group died before day 28 (*P* = 1.000) and were not evaluable for chimerism analysis on day 28. The remaining patients were analyzed regarding chimerism on day 28. The TBI group and non-TBI group showed no differences regarding full donor chimerism on day 28 (TBI 90.9%, non-TBI 93.4%; *P* = 0.475).

### Time-to-event analyses for all endpoints

The causes of death (including mortality caused by SMs) after TBI-based conditioning and non-TBI-based conditioning are listed in Table 3. Relapse of AML was the most frequent cause of death for the overall population (47.6%), followed by NRM-GVHD (22.2%) and NRM-infectious deaths (19.0%). Two patients died due to SMs after TBI-based conditioning and 3 patients after non-TBI-based conditioning (Table 3).

**Table 3:** Causes of death including mortality caused by secondary malignancies after TBI-based conditioning and non-TBI-based conditioning

Cause of death	Total (%) (*n* = 189)	TBI-based conditioning (%) (*n* = 55)	Non-TBI-based conditioning (FBM, FTM, FM) (%) (*n* = 134)	*P*-value
AML	90 (47.6%)	37 (67.3%)	53 (39.6%)	0.006
NRM, GVHD	42 (22.2%)	7 (12.7%)	35 (26.1%)	
NRM, infection	36 (19.0%)	6 (10.9%)	30 (22.4%)	
NRM, other causes	21 (11.1%)	5 (9.1%)*	16 (11.9%)**	

*AML*, acute myeloid leukemia; *NRM*, non-relapse mortality; *GVHD*, graft-versus-host disease; *TBI*, total body irradiation; *FBM*, fludarabine, BCNU, melphalan; *FTM*, fludarabine, thiotepa, melphalan; *FM*, fludarabine, melphalan; *NRM*, other causes of death include secondary malignancies, graft failure, accidents, suicides, hemorrhage, or thrombosis.

*Two patients died due to secondary malignancies after TBI-based conditioning. A male patient (non-smoker) developed a primary metastatic prostate cancer 149.2 months after allogeneic hematopoietic stem cell transplantation. He died 42.6 months after diagnosis of the prostate cancer due to the secondary malignancy. A woman (non-smoker) developed a metastatic undifferentiated pleomorphic sarcoma of the lower extremity with lymph node metastases 10.6 months after allogeneic hematopoietic stem cell transplantation. It was assumed that the sarcoma was present at time of allogeneic hematopoietic stem cell transplantation. She died 8.9 months after diagnosis of the sarcoma due to the secondary malignancy.

**Three patients died due to secondary malignancies after non-TBI-based conditioning. A male patient (non-smoker) developed a squamous cell cancer of the esophagus 3.2 years after allogeneic hematopoietic stem cell transplantation. He died 0.53 years after diagnosis of his secondary malignancy due to the secondary malignancy. A female patient (smoker) developed a non-small cell squamous cell lung cancer 7.9 years following allogeneic hematopoietic stem cell transplantation. The patient died 0.78 years after diagnosis of the lung cancer. A male patient (smoker) developed a non-small cell adeno lung cancer with pleural affection 2.8 years following allogeneic hematopoietic stem cell transplantation. He died 0.5 months after diagnosis of his lung cancer due to the secondary malignancy

Cumulative incidence rates of clinical outcomes comparing TBI vs. non-TBI on specific time points are shown in Table 4.

**Table 4:** Cumulative incidence rates of clinical outcomes on specific time points

	Total (*n* = 339)	TBI-based conditioning (*n* = 91)	Non-TBI-based conditioning (FTM, FBM, FM) (*n* = 248)	*P*-value
NRM				
100-day	8% (95% CI, 5–11)	6% (95% CI, 2–12)	9% (95% CI, 5–12)	0.320
2-year	23% (95% CI, 19–28)	15% (95% CI, 9–24)	26% (95% CI, 21–32)	0.021
5-year	29% (95% CI, 24–34)	17% (95% CI, 10–25)	33% (95% CI, 27–40)	< 0.001
CIR				
2-year	27% (95% CI, 22–31)	36% (95% CI, 27–46)	23% (95% CI, 18–28)	0.020
5-year	33% (95% CI, 27–38)	42% (95% CI, 32–52)	29% (95% CI, 23–35)	0.030
Grade II–IV aGVHD				
100-day	41% (95% CI, 35–46)	43% (95% CI, 33–53)	40% (95% CI, 34–46)	0.627
cGVHD				
2-year	34% (95% CI, 29–39)	33% (95% CI, 24–43)	35% (95% CI, 29–41)	0.784
5-year	35% (95% CI, 30–41)	34% (95% CI, 24–44)	36% (95% CI, 30–42)	0.789
OS				
2-year	42% (95% CI, 37–47)	43% (95% CI, 33–53)	42% (95% CI, 35–48)	0.849
5-year	55% (95% CI, 49–60)	54% (95% CI, 43–64)	55% (95% CI, 48–62)	0.855
PFS				
2-year	50% (95% CI, 44–55)	52% (95% CI, 41–61)	49% (95% CI, 43–55)	0.685
5-year	61% (95% CI, 55–66)	58% (95% CI, 47–68)	62% (95% CI, 55–68)	0.531

*FBM*, fludarabine, BCNU, melphalan; *FTM*, fludarabine, thiotepa, melphalan; *FM*, fludarabine, melphalan; *NRM*, non-relapse mortality; *CIR*, cumulative incidence of relapse; *aGVHD*, acute graft-versus-host disease; *cGVHD*, chronic graft-versus-host disease; *OS*, overall survival; *PFS*, progression free survival

**Fig. 1:**
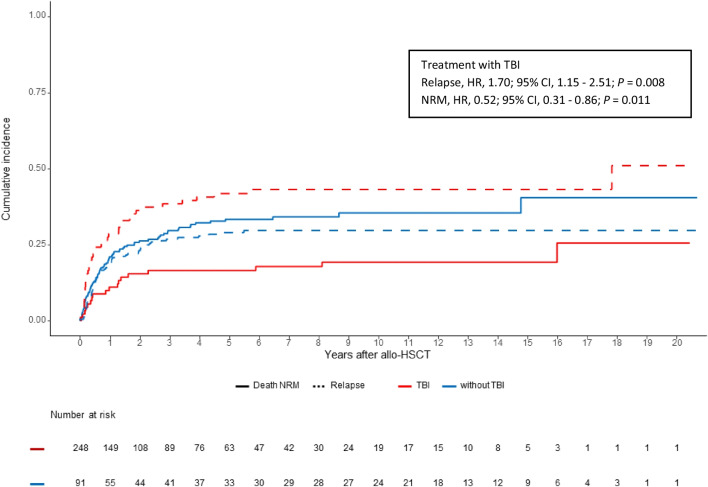
Transplantation outcomes after TBI-based conditioning and non-TBI-based conditioning (FBM, FTM, FM): estimates of the cumulative incidences of non-relapse mortality (NRM, solid line) and relapse (CIR, dotted line) in a competing risk setting (TBI vs. non-TBI)

Figure 1 shows the estimates of the cumulative incidences of NRM and relapse (CIR) in a competing risk setting for both treatment groups (TBI vs. non-TBI).

**Fig. 2:**
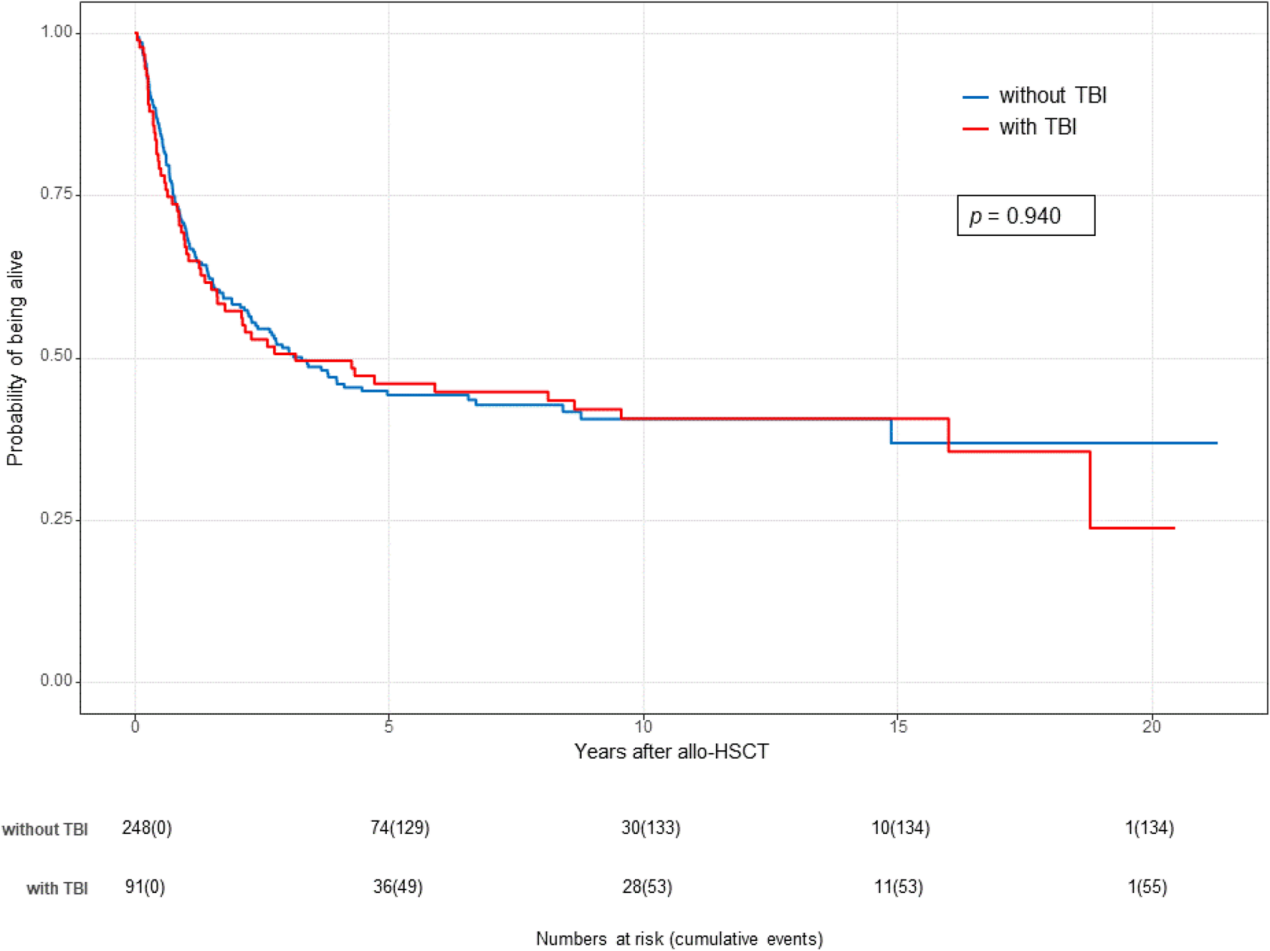
Transplantation outcomes after TBI-based conditioning and non-TBI-based conditioning (FBM, FTM, FM): Kaplan–Meier estimates of overall survival (OS) by treatment group (TBI vs. non-TBI)

**Fig. 3:**
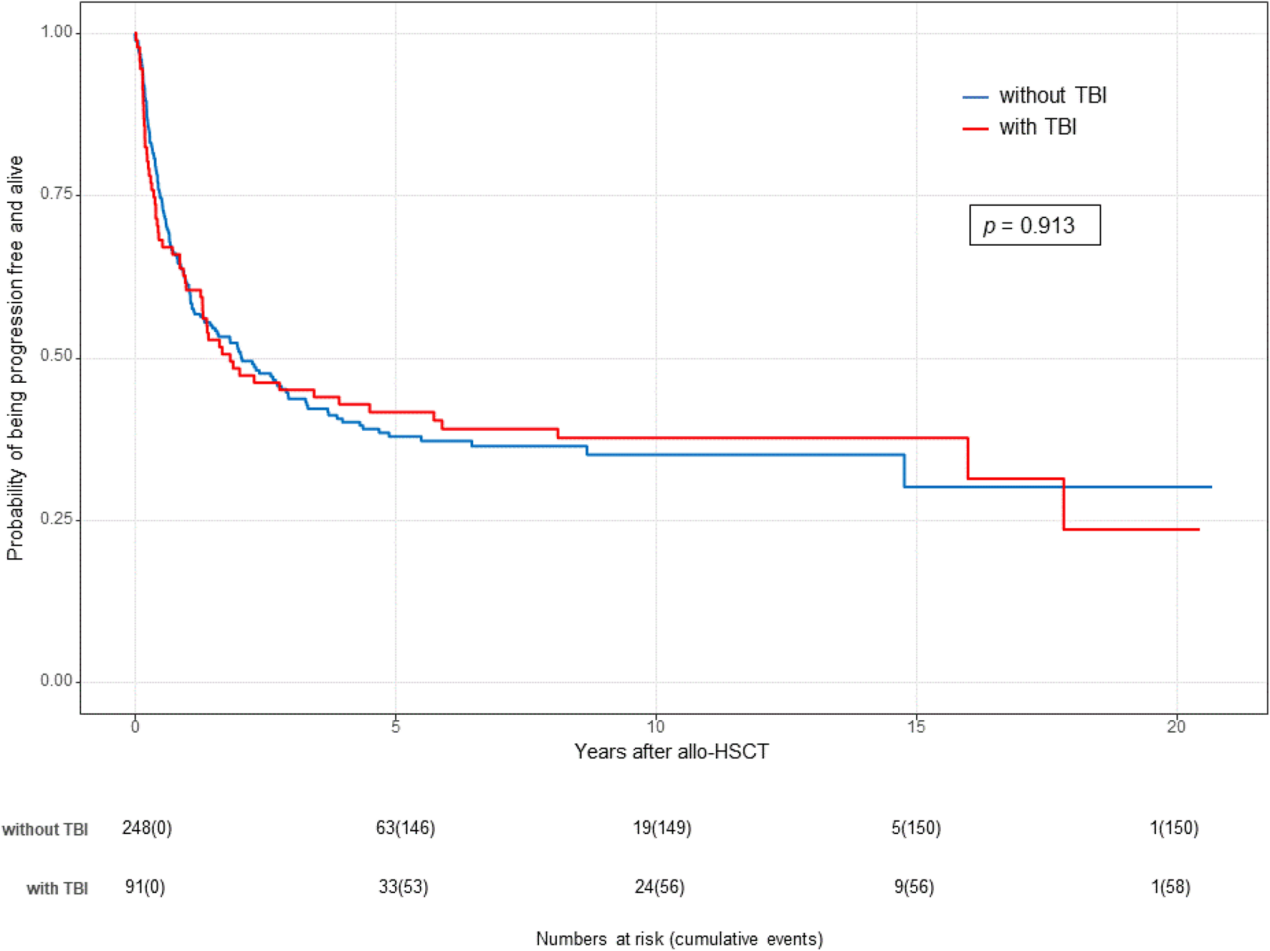
Transplantation outcomes after TBI-based conditioning and non-TBI-based conditioning (FBM, FTM, FM): Kaplan–Meier estimates of progression-free survival (PFS) by treatment group (TBI vs. non-TBI)

Figure 2 and Fig. 3 show the Kaplan–Meier estimates of OS and PFS for both treatment groups (TBI vs. non-TBI). OS and PFS were similar for both treatment groups.

Table 5 summarizes the results of the univariable and multivariable analysis of clinical outcomes. TBI was no risk factor for NRM, CIR, PFS and OS in the multivariable regression models. Adverse ELN risk stratification translated into a higher CIR (HR, 2.48; 95% CI, 1.21–5.08) and a decreased chance of PFS (HR, 2.10; 95% CI, 1.33–3.33) and OS (HR, 1.81; 95% CI, 1.12–2.91) compared to favorable ELN risk stratification. Furthermore, advanced disease status (> second complete remission, CR2) negatively affected CIR (HR, 1.99; 95% CI, 1.18–3.36), NRM (HR, 2.52; 95% CI 1.46–4.35), PFS (HR, 2.95; 95% CI, 2.04–4.26), and OS (HR, 3.61; 95% CI, 2.44–5.33) compared to transplantation in first complete remission (CR1). Older patient age at the time of 1st allo-HCST translated into a higher risk of NRM (HR, 1.03; 95% CI, 1.01–1.06) and a lower chance of PFS (HR, 1.02; 95% CI, 1.00–1.03) and OS (HR, 1.02; 95% CI, 1.00–1.04). Patients with HCT-CI scores of 3 had a lower PFS (HR, 1.63; 95% CI, 1.13–2.35) and OS (HR, 1.49; 95% CI, 1.01–2.18) compared to patients with HCT-CI scores of 0. A female donor for a male recipient was a poor prognostic factor for NRM (HR, 2.15; 95% CI, 1.25–3.72) compared to other gender combinations. Patients with a Karnofsky performance status ≥ 80 had a higher chance of OS (HR, 0.60; 95% CI, 0.40–0.90) and PFS (HR, 0.55; 95% CI, 0.37–0.82) compared to patients with a Karnofsky performance status < 80 (Table 5).

**Table 5:** Univariable and multivariable analysis of clinical outcomes

	Relapse, competing NRM		NRM, competing relapse		PFS		OS		Chronic GVHD, competing relapse or death without cGVHD		Grade II–IV acute GVHD, competing relapse or death without aGVHD	
	HR (95% CI)	*P*-value	HR (95% CI)	*P*-value	HR (95% CI)	*P*-value	HR (95% CI)	*P*-value	HR (95% CI)	*P*-value	HR (95% CI)	*P*-value
Univariable model												
Treatment with non-TBI-based regimen* (reference)	1		1		1		1		1		1	
Treatment with TBI-based regimen	1.70 (1.15–2.51)	0.008	0.52 (0.31–0.86)	0.011	0.98 (0.72–1.33)	0.914	1.01 (0.74–1.39)	0.939	1.00 (0.68–1.48)	0.998	1.17 (0.82–1.67)	0.391
Multivariable model												
Treatment with non-TBI-based regimen* (reference)	1		1		1		1		1		1	
Treatment with TBI-based regimen	1.40 (0.70–2.82)	0.345	0.72 (0.34–1.51)	0.380	1.07 (0.66–1.74)	0.791	1.10 (0.68–1.80)	0.691	1.12 (0.61–2.07)	0.718	1.18 (0.70–2.01)	0.529
2017 ELN genetic risk stratification												
Favorable (reference)	1		1		1		1		1		1	
Intermediate	1.11 (0.53–2.33)	0.787	1.36 (0.68–2.71)	0.381	1.25 (0.78–2.01)	0.361	1.13 (0.69–1.85)	0.631	1.20 (0.66–2.16)	0.549	1.06 (0.62–1.80)	0.840
Adverse	2.48 (1.21–5.08)	0.013	1.05 (0.52–2.10)	0.892	2.10 (1.33–3.33)	0.002	1.81 (1.12–2.91)	0.015	0.95 (0.53–1.72)	0.875	1.10 (0.65–1.87)	0.717
Secondary AML	0.89 (0.56–1.42)	0.614	1.35 (0.87–2.09)	0.181	1.05 (0.77–1.43)	0.764	1.18 (0.86–1.64)	0.306	1.34 (0.86–2.08)	0.190	0.92 (0.62–1.35)	0.659
Disease status at the time of 1st allogeneic hematopoietic stem cell transplantation												
First complete remission (CR1) (reference)	1		1		1		1		1		1	
CR2, first partial remission (PR1)	1.53 (0.95–2.46)	0.082	1.60 (0.93–2.75)	0.087	1.73 (1.22–2.47)	0.002	1.77 (1.21–2.60)	0.003	0.94 (0.60–1.47)	0.790	1.46 (0.96–2.22)	0.078
> CR2, refractory or active AML	1.99 (1.18–3.36)	0.010	2.52 (1.46–4.35)	0.001	2.95 (2.04–4.26)	< 0.001	3.61 (2.44–5.33)	< 0.001	0.32 (0.17–0.61)	0.001	1.67 (1.06–2.62)	0.026
Hematopoietic cell transplantation-comorbidity index (HCT-CI)												
Score 0 (reference)	1		1		1		1		1		1	
Score 1–2	1.14 (0.71–1.84)	0.594	1.38 (0.81–2.36)	0.232	1.37 (0.96–1.95)	0.084	1.34 (0.92–1.95)	0.126	0.95 (0.60–1.52)	0.837	0.86 (0.56–1.32)	0.485
Score ≥ 3	1.34 (0.80–2.27)	0.266	1.37 (0.80–2.34)	0.255	1.63 (1.13–2.35)	0.009	1.49 (1.01–2.18)	0.043	1.05 (0.63–1.73)	0.855	1.08 (0.72–1.63)	0.710
Patient age	1.00 (0.98–1.02)	0.700	1.03 (1.01–1.06)	0.005	1.02 (1.00–1.03)	0.023	1.02 (1.00–1.04)	0.013	0.98 (0.96–0.99)	0.007	1.01 (0.99–1.03)	0.239
Karnofsky performance score ≥ 80	0.81 (0.44–1.50)	0.513	0.71 (0.38–1.34)	0.294	0.55 (0.37–0.82)	0.003	0.60 (0.40–0.90)	0.013	1.27 (0.64–2.54)	0.493	0.96 (0.60–1.53)	0.860
Donor type												
Matched sibling donor(reference)	1		1		1		1		1		1	
Matched unrelated donor	0.62 (0.33–1.16)	0.131	1.29 (0.65–2.54)	0.463	0.79 (0.51–1.24)	0.308	0.81 (0.51–1.28)	0.358	1.03 (0.60–1.76)	0.916	1.38 (0.81–2.33)	0.235
Mismatched unrelated donor	0.77 (0.35–1.71)	0.527	1.21 (0.57–2.54)	0.622	0.97 (0.58–1.63)	0.911	0.95 (0.55–1.65)	0.858	1.03 (0.51–2.10)	0.930	1.22 (0.64–2.32)	0.540
Haploidentical, mismatched related	0.36 (0.04–2.88)	0.333	1.08 (0.18–6.50)	0.936	0.49 (0.14–1.70)	0.258	0.58 (0.16–2.05)	0.397	0.68 (0.12–3.69)	0.651	1.71 (0.42–7.04)	0.455
Stem cell source bone marrow	1.13 (0.54–2.38)	0.742	1.10 (0.32–3.81)	0.876	1.09 (0.59–2.04)	0.782	1.23 (0.65–2.31)	0.527	0.51 (0.17–1.57)	0.242	0.86 (0.37–2.04)	0.738
Female donor to male recipient	0.66 (0.36–1.22)	0.183	2.15 (1.25–3.72)	0.006	1.15 (0.78–1.71)	0.473	1.45 (0.98–2.15)	0.065	1.42 (0.87–2.32)	0.157	1.25 (0.80–1.96)	0.330
Donor age	0.99 (0.97–1.01)	0.458	1.01 (0.99–1.03)	0.220	1.00 (0.99–1.01)	0.972	1.00 (0.99–1.02)	0.804	1.00 (0.99–1.02)	0.582	1.01 (1.00–1.03)	0.114
Donor/recipient cytomegalic virus serostatus												
Negative/negative (reference)	1		1		1		1		1		1	
Negative/positive	0.88 (0.50–1.56)	0.664	1.77 (0.93–3.37)	0.080	1.13 (0.74–1.71)	0.571	1.14 (0.74–1.76)	0.559	1.09 (0.60–1.95)	0.783	1.31 (0.82–2.10)	0.257
Positive/positive	0.75 (0.45–1.24)	0.262	1.74 (1.00–3.02)	0.049	1.00 (0.70–1.42)	0.979	1.18 (0.82–1.72)	0.376	0.82 (0.50–1.33)	0.415	1.28 (0.83–1.96)	0.263
Positive/negative	0.73 (0.36–1.50)	0.398	1.68 (0.86–3.29)	0.132	1.00 (0.62–1.61)	0.993	0.94 (0.56–1.57)	0.818	0.67 (0.36–1.25)	0.209	0.90 (0.50–1.63)	0.736
Antithymocyte globulin	1.04 (0.54–2.00)	0.907	0.91 (0.53–1.55)	0.722	0.94 (0.62–1.41)	0.760	0.89 (0.58–1.35)	0.573	0.63 (0.38–1.06)	0.081	0.68 (0.44–1.04)	0.078
Reduced intensity conditioning	0.86 (0.40–1.84)	0.697	1.05 (0.35–3.10)	0.935	0.75 (0.41–1.38)	0.355	0.76 (0.41–1.41)	0.383	1.88 (0.89–3.96)	0.099	0.81 (0.41–1.60)	0.537

*NRM*, non-relapse mortality; *PFS*, progression free survival; *OS*, overall survival; *GVHD*, graft-versus-host disease; *non-TBI-based regimen: *FBM*, fludarabine, BCNU, melphalan; *FTM*, fludarabine, thiotepa, melphalan; *FM*, fludarabine, melphalan

TBI-based conditioning was no risk factor for grade II–IV aGVHD and cGVHD. Patient age at the time of transplantation had a significant impact on cGVHD (HR, 0.98; 95% CI, 0.96–0.99). Advanced disease at the time of 1st transplantation (> CR2, refractory/active AML) was associated with a lower risk for cGVHD (HR, 0.32; 95% CI, 0.17–0.61), but a higher risk for grade II–IV aGVHD (HR, 1.67; 95% CI, 1.06–2.62) compared to transplantations in CR1 (Table 5).

## Discussion

This retrospective study analyzed the long-term outcome in patients after TBI-based and non-TBI-based conditioning focusing on AML patients and 1st allo-HSCT. The cumulative incidences of 2-year and 5-year NRM after TBI-based conditioning were 15% and 17%, respectively. Similar NRM after TBI-based conditioning has been observed by some multicenter studies containing TBI [11, 12]. Our findings indicate that long-term NRM is low after modern TBI-based conditioning and long-term outcome appears to be identical compared to non-TBI-based regimens. TBI-based conditioning was no risk factor for NRM including mortality caused by secondary malignancies which is one of the major concerns in long-term survivors after allo-HSCT. Our results are consistent with the results of Morsink et al. [13]. Morsink et al. [13] compared the results of high-dose (HD)-TBI-based (TBI 12 Gy or TBI 13.2 Gy) and non-HD-TBI-based MAC (busulfan/cyclophosphamide, busulfan/fludarabine, treosulfan/fludarabine ± TBI 2 Gy) among adults with AML who underwent a first allo-HSCT in the first or second morphologic remission. HD-TBI was not associated with different outcomes (relapse, RFS, OS and NRM) compared to non-HD-TBI conditioning.

TBI-based and non-TBI-based regimens had similar cumulative incidences of aGVHD and cGVHD; thus, TBI was no risk factor for GVHD. Our results show that the addition of ATG had no negative effects on CIR, NRM, and OS in the multivariable model [14]. In summary, relapse of AML remains the prime cause of transplant failure independent of the conditioning regimen.

Our study was underpowered to answer the question of whether patients had disadvantages regarding cataracts, thyroid diseases, or pulmonary complications after TBI-based conditioning, or to draw a firm conclusion of the different TBI-based regimens (8 vs. 12 Gy) regarding efficacy. Nevertheless, the multivariable analysis did not associate TBI-based regimens with any of the outcome variables analyzed.

Unfortunately, literature shows a considerable variability in planning, prescription, and treatment with TBI [15]. Variations involve treatment techniques, methods of fractionation, dose rates, methods of dosimetry, and lung shielding. Dose rates can vary from 2.25 to 37.5 cGy/min and photon energy from 6 to 25 MV [15]. Overall literature data support for the use of lung shielding and dose rates of 7.5 cGy/min or less rather than 15 cGy/min, as well as a twice-daily fractionation to reduce pulmonary complications and toxicity to normal tissue [16, 17]. Reasons for our favorable long-term NRM after TBI-based conditioning may be the superior lung shielding in case of doses of > 8 Gy and the consistent average dose rate of 4 cGy/min to the total body, as well as the twice-daily fractionation. However, the improved NRM is not solely based on optimized TBI technologies, but also results from a selection bias, as TBI-conditioned patients were overall younger and in better health condition. This risk-based patient selection by the transplant physicians was defined in institutional guidelines and in line with recommendations and clinical practice at most transplant centers. New TBI technologies, such as total marrow irradiation (TMI) in combination with volumetric modulated arc therapy (VMAT), may further improve results by delivering targeted forms of TBI [18, 19].

This study is limited by its retrospective nature and the comparatively small number of patients conditioned with TBI. Moreover, the selection bias to treat younger patients with fewer comorbidities and high-risk cytogenetics with TBI, and older patients with non-TBI-based regimens, prohibits matched pair analyses. The primary strength of the present study is the consistent delivery of TBI over 20 years with no major variations of other variables. Additionally, data completeness was 100% through active monitoring of all transplanted patients.

## Conclusions

The findings indicate that long-term NRM is low after modern TBI-based conditioning and outcome appears to be identical compared to non-TBI-based regimens. Therefore, TBI-based conditioning can be considered part of standard of care for AML patients eligible for 1st allo-HSCT.

## Data Availability

The datasets analyzed during the current study are available from the corresponding author on reasonable request.
